# Optimization of Antimicrobial Concentrations in an Agar-Free, Low-Cost, Wheat Germ-Based Diet for Mass Rearing of *Drosophila suzukii*

**DOI:** 10.3390/insects17090960

**Published:** 2026-09-16

**Authors:** Jaime González-Cabrera, César Gálvez, Yadira Contreras-Bermúdez, Jorge A. Sánchez-González, Uriel Carlos Cruz Mendez, Ricardo A. Toledo-Hernández

**Affiliations:** 1 Departamento de Control Biológico, Centro Nacional de Referencia de Control Biológico, Centro Nacional de Referencia Fitosanitaria-Servicio Nacional de Sanidad, Inocuidad y Calidad Agroalimentaria, Tecomán 28110, Colima, Mexico; jgonz017@ucr.edu (J.G.-C.); cesarbiotecnologia@gmail.com (C.G.); yady_23y@hotmail.com (Y.C.-B.); j_asg2@hotmail.com (J.A.S.-G.); 2 Facultad de Ciencias Biológicas y Agropecuarias, Universidad de Colima, Km. 40 Autopista Colima-Manzanillo, Tecomán 28934, Colima, Mexico; ccruz11@ucol.mx; 3 Departamento de Ecología de Artrópodos y Manejo de Plagas, El Colegio de la Frontera Sur (ECOSUR), Carretera Antiguo Aeropuerto Km. 2.5, Tapachula 30700, Chiapas, Mexico; 4 Departamento de Sanidad Vegetal, Plantas de Navarra, S.A. (PLANASA), Fuente Olmo de Fuentidueñas, 40359 Segovia, Castilla y León, Spain

**Keywords:** spotted-wing drosophila, augmentative biological control, mass rearing, artificial diet, Taguchi method, antimicrobial optimization

## Abstract

The bottleneck for the mass production of *Trichopria drosophilae* Perkins (Hymenoptera: Diapriidae), the principal parasitoid of the *Drosophila suzukii* (Matsumura) (Diptera: Drosophilidae), is the lack of efficient mass-rearing methods. Here, we optimized the concentrations of five antimicrobials (each at three concentration levels) in an agar-free, low-cost, wheat germ-based diet using a Taguchi orthogonal array design (L27 = 3^5^). The optimal formulation (as determined indirectly through insect-rearing performance) consisted of 1.2 g L^−1^ of sodium benzoate, 1.2 g L^−1^ of propyl paraben, 0.3 g L^−1^ of potassium sorbate, 0 g L^−1^ of sodium propionate, and 0 g L^−1^ of triclosan. In the Taguchi screening, the best-performing treatment produced approximately 4.9 times as many pupae as the antimicrobial-free control. Subsequent validation confirmed that both the best-performing treatment and the optimized formulation significantly increased pupal production relative to the internal antimicrobial-free control. Furthermore, the formulation reduced the number of antimicrobial compounds required, simplifying diet preparation and lowering production costs. Additionally, because the diet does not contain agar and requires only basic equipment and simple procedures, it represents a practical and scalable alternative for the mass production of *D. suzukii* and its parasitoid *T. drosophilae*. This optimized, low-cost formulation is expected to facilitate the scalable mass rearing of *T. drosophilae*, thereby promoting its adoption as a practical and accessible biological control strategy against *D. suzukii*, especially in resource-limited berry-producing regions.

## Simple Summary

In berry crops, the spotted wing fruit fly (*Drosophila suzukii*) is a major pest that causes significant economic losses. The pupal parasitoid *Trichopria drosophilae* is a promising biological control agent. However, mass production of this parasitoid is limited by microbial contamination during rearing of its host on artificial diets. At the same time, antimicrobials may negatively affect host development when inappropriate compounds are used. In this study, we optimized the antimicrobial formulation of a low-cost, agar-free, wheat germ-based diet using the Taguchi method. Five antimicrobial agents were evaluated indirectly through insect-rearing performance at three concentration levels each to identify the best combination for enhancing *D. suzukii* production. The optimal formulation consisted of sodium benzoate and propyl paraben (1.2 g L^−1^ each) and 0.3 g L^−1^ of potassium sorbate, while sodium propionate and triclosan were not required. In the Taguchi screening, the best-performing treatment produced approximately 4.9 times as many pupae as the antimicrobial-free control. Validation further confirmed that both the best-performing treatment and the optimized formulation significantly improved pupal production.

## 1. Introduction

The spotted-wing drosophila, *Drosophila suzukii* (Matsumura) (Diptera: Drosophilidae), is one of the most destructive invasive pests affecting soft-skinned fruit crops worldwide. Infestations by this species result in substantial yield losses and increased production costs across major berry-producing regions [1]. In contrast to most drosophilid species, female *D. suzukii* possess a serrated ovipositor that allows oviposition in ripening and ripe fruits, like berries, cherries, grapes, and other thin-skinned crops. Consequently, larval development within the fruit causes direct feeding damage, accelerates fruit decay, and facilitates secondary microbial infections [1,2,3,4,5]. These biological traits complicate population management and represent a significant threat to the economic sustainability of fruit production systems.

The economic impact of *D. suzukii* is particularly severe in high-value berry crops such as blueberries, strawberries, and raspberries, where infestations can result in substantial revenue losses. These economic costs stem not only from direct yield reduction but also from the implementation of intensive and labor-demanding management practices [6,7,8], such as growth regulators, flowering stimulants, repellents, exclusion nets, complete crop covers, mulches, and volatile-baited traps [1,8,9,10,11]. Despite the integration of these approaches, effective and sustainable management of *D. suzukii* remains a major challenge for fruit growers [11].

Since its first detection in Mexico in 2011, *D. suzukii* has become a major threat to the national berry industry [12,13]. In affected regions worldwide, infestations have been associated with yield losses of up to 39% in conventional production systems and up to 100% in organic [6,10,11,14,15,16]. Current management in Mexico depends largely on chemical insecticides and cultural control practices; however, these measures are costly, labor-intensive, and often insufficient to achieve satisfactory suppression of pest populations [1]. Moreover, their long-term sustainability is limited by economic and environmental concerns. Therefore, the identification and implementation of more effective and sustainable management alternatives remain critical for mitigating the impact of *D. suzukii* on fruit production [11]. Among biological control options available for *D. suzukii*, the pupal parasitoid *Trichopria drosophilae* Perkins (Hymenoptera: Diapriidae) has emerged as one of the most promising agents [17,18]. This parasitoid is widely distributed worldwide, including Mexico, and exhibits remarkable adaptability to diverse environmental conditions, tolerating temperatures ranging from 5 to 35 °C [19,20]. Moreover, it shows a strong preference for *D. suzukii* and has achieved high parasitism rates in laboratory, greenhouse, and field studies [17,18,21,22,23,24]. Despite its presence in Mexico, populations of *T. drosophilae* occur at very low densities in berry-producing systems, limiting their contribution to pest suppression [25]. Consequently, although this species has considerable potential as a biological control agent, it is not yet used commercially as either a primary or supplementary control strategy. The major constraint to its large-scale implementation remains the lack of efficient and economically viable mass-rearing protocols [24].

Traditional artificial diets used for the mass rearing of *D. suzukii* and *D. melanogaster* pupae, which serve as hosts for the mass production of *T. drosophilae*, often require costly ingredients such as agar, specialized laboratory infrastructure (e.g., laminar-flow hoods), and stringent aseptic conditions to prevent microbial contamination [20]. These requirements substantially increase production costs and limit the implementation of mass-rearing programs, particularly in small-scale facilities and resource-limited regions such as Mexico. To overcome these constraints, a low-cost wheat germ-based artificial diet for the mass rearing of *T. drosophilae* was developed in Mexico in 2015 [13,26,27]. This diet was specifically designed to reduce production costs by eliminating expensive gelling agents, simplifying preparation through a brief boiling process, and avoiding the need for sophisticated containment infrastructure. Instead, only temperature and relative humidity are regulated using commercially available household equipment [13,27]. Subsequent improvements to the nutritional composition of the diet significantly enhanced the reproductive performance of both the *D. suzukii* host and the *T. drosophilae* parasitoid [26,28].

Despite these advances, microbial contamination remains a major operational challenge in the mass production system of the low-cost wheat germ-based artificial diet. Opportunistic fungi and bacteria are among the most common contaminants in artificial insectary environments. Their proliferation can reduce diet palatability, deplete essential nutrients, generate toxic metabolites, and adversely affect insect development and survival, resulting in pupal losses approaching 50% under mass-rearing conditions [28]. Therefore, effective suppression of microbial contaminants is essential to ensure the efficiency, reliability, and scalability of the production system.

The development of an effective antimicrobial matrix requires compounds capable of suppressing a broad spectrum of microorganisms without adversely affecting the biological performance of either the host insect or its parasitoid [29,30]. To improve *D. suzukii* rearing efficiency and maximize *T. drosophilae* production, we evaluated a multi-component antimicrobial system consisting of sodium benzoate, propyl paraben, potassium sorbate, sodium propionate, and triclosan.

These compounds were selected because of their complementary antimicrobial spectra against fungi, bacteria, and yeasts, as well as their distinct modes of action, including membrane disruption and inhibition of key metabolic pathways [29,31,32].

A full factorial evaluation of those five antimicrobial compounds at three concentration levels would require 243 treatment combinations (3^5^), making experimentation costly and time-consuming. Therefore, we employed a Taguchi L27 (3^5^) orthogonal array design, an efficient statistical approach that enables the simultaneous evaluation of multiple factors and their interactions with a substantially reduced treatment load [33,34]. The objectives of this study were to optimize the antimicrobial composition of a wheat germ-based artificial diet using the Taguchi method, with antimicrobial effectiveness evaluated indirectly through *D. suzukii* rearing performance, and to assess its potential contribution to the mass production of *T. drosophilae* for biological control and integrated pest management (IPM) programs targeting *D. suzukii* in berry-producing regions.

## 2. Materials and Methods

### 2.1. Place of Study and Drosophila suzukii Colony History

All experiments were conducted at the Department of Entomophagous Insects of the National Reference Center for Biological Control (CNRCB, for its acronym in Spanish), which is affiliated with the General Directorate of Plant Health (DGSV, for its acronym in Spanish) of the National Service for Agro-alimentary Health, Safety and Quality (SENASICA, for its acronym in Spanish), Tecoman, Colima, Mexico.

The *D. suzukii* colony used in this study was established from adults collected from blackberry plants, *Rubus fruticosus* L. (Rosaceae), in Colima (19°24′28.8″ N and 103°35′34.8″ O), Mexico, in 2013 [35]. Since its establishment, the colony has been continuously maintained under controlled laboratory conditions at 23 ± 1 °C, 40 ± 5% relative humidity (RH), and a natural photoperiod ranging from 11:13 to 13:11 h (L:D) on a wheat germ-based artificial diet (see 2.2 for diet specification). Adults were maintained in screened cages (40 × 40 × 40 cm). At the time of the experiments, the colony had been maintained for approximately 130 generations.

To maintain genetic diversity and reduce the effects of inbreeding and laboratory adaptation, 300–400 field-collected adults were randomly introduced annually into the colony. Unless otherwise indicated, all experiments were conducted under the same environmental conditions (23 °C and 40% RH, and a natural photoperiod ranging from 11:13 to 13:11 h (L:D), typical for the annual fluctuations in Tecomán, Colima). Each treatment was replicated independently three times.

### 2.2. Wheat Germ Diet History and Preparation

The artificial diet optimized in this study (hereafter referred to as “standard wheat germ diet”) is primarily composed of wheat germ and uses sodium benzoate as the principal antimicrobial preservative, along with smaller amounts of potassium sorbate, propyl paraben, sodium propionate, and triclosan. This diet was derived from the mass-rearing formulation originally developed for *Anastrepha ludens* Loew (Diptera: Tephritidae) [36] and was subsequently modified by González-Cabrera et al. [13,26,28] (2018a, 2018b, 2023). The resulting formulation was adopted for routine colony maintenance in August 2019.

The standard wheat germ diet was prepared by mixing 1 L of purified water, 12.5 g of brewer’s yeast, 60 g of maize flour, 83.3 g of raw wheat germ, 75 g of table sugar, 1.33 g of potassium sorbate, 1.33 g of sodium propionate, 13.3 g of sodium benzoate (El Harinero, Colima, Mexico), and 66.6 g of wheat bran. The mixture was heated to boiling and continuously stirred for approximately 7 min until a homogeneous slurry was obtained. After cooling at room temperature for 7 min, 2.7 g of 1 M hydrochloric acid were added, as well as 1.33 g of propyl paraben and 0.16 g of triclosan (Drogueria Cosmopolita, Naucalpan de Juárez, Mexico) dissolved in 20 mL of 95% ethyl alcohol, and thoroughly mixed, resulting in a final diet pH of 4.6 ± 0.1. The final mass of slurry was approximately 900 g, from which approximately 70 g of the prepared diet was dispensed into each of eight Petri dishes (8 cm diameter × 1.4 cm height), which were immediately used as oviposition substrate [27].

Rearing of *D. suzukii* on the wheat germ diet was carried out according to the methodology described by Mendoza-Ceballos et al. [27] (2018) and Gonzalez-Cabrera et al. [28] (2023). Eight Petri dishes containing the diet were placed inside each reproduction cage (40 × 40 × 40 cm) consisting of a plastic frame covered with organza fabric and an external layer of Agribón fabric (Bonlam S.A. de C.V., San Luis Potosí, Mexico). A total of two cages were maintained simultaneously to sustain the colony. Each cage contained approximately 400 adult flies (≥4 days old) and a 24.8 mL plastic cup fitted with a cotton wick soaked in diluted honey solution (2:3 honey:water ratio) as a food source. The honey solution was replaced twice weekly to ensure continuous food availability and prevent microbial contamination.

After a 48 h oviposition period, the Petri dishes were removed from the cages and individually transferred to plastic trays (17 cm diameter × 8 cm high; R10^®^ Reyma, Guanajuato, Mexico) to allow embryonic development of the eggs and larval development. Pupation occurred approximately 8–10 days later. The contents of each Petri dish were then submerged in a 10 L container filled with tap water and gently agitated to separate the pupae from the diet. Floating pupae were collected using a plastic sieve (7 cm diameter), drained on paper towels for 10 min, air-dried, and transferred to clean Petri dishes for adult emergence.

This rearing procedure was repeated every three days. A portion of the resulting pupae were used as hosts for parasitoid production, whereas the remaining pupae were retained for maintenance of the laboratory colony.

### 2.3. Optimization of Antimicrobial Concentrations in the Wheat Germ Diet

A Taguchi L27 orthogonal array (3^5^) was used to identify the optimal combination of five antimicrobial agents (factors) [37], each evaluated at three concentrations (hereafter referred to as levels L1, L2, and L3), for controlling microbial contamination in the *D. suzukii* diet [33,34].

**Figure 1:**
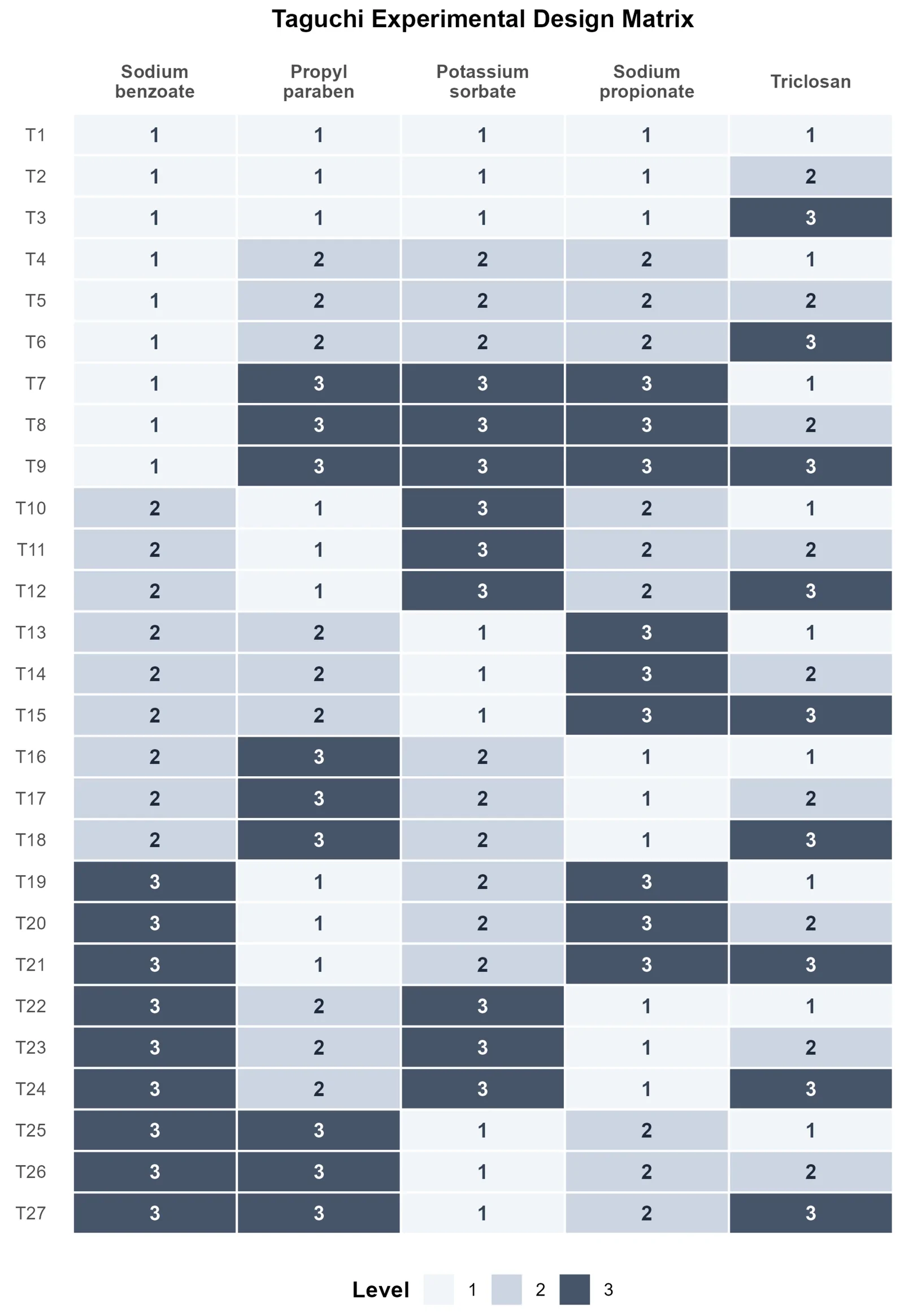
Experimental design matrix of the Taguchi L27 (3^5^) orthogonal array was used to evaluate the effects of five antimicrobial compounds in the artificial diet for *Drosophila suzukii*. Treatments (T1–T27) consisted of different combinations of sodium benzoate, propyl paraben, potassium sorbate, sodium propionate, and triclosan at three concentration levels. Levels 1, 2, and 3 corresponded to concentrations of 0 g, 0.3 ± 0.1 g, and 1.2 ± 0.1 g, respectively, for all antimicrobials except triclosan, for which concentrations were 0 g, 0.075 ± 0.01 g, and 0.15 ± 0.01 g.

The antimicrobial agents were selected based on two criteria: (i) their commercial availability, as they can be readily obtained without special authorization, and (ii) their diverse modes of action (Table 1), which may help to reduce the risk of microbial resistance development [29,30,38]. The antimicrobials evaluated were sodium benzoate, potassium sorbate, propyl paraben, sodium propionate, and triclosan. The tested concentrations for the first four antimicrobials were 0 g, 0.3 ± 0.1 g, and 1.2 ± 0.1 g per batch of the standard wheat-germ diet, whose ingredients and quantities were described previously. Likewise, Triclosan was evaluated at 0 g, 0.075 ± 0.01 g, and 0.15 ± 0.01 g per batch of the standard diet (Figure 1). Concentration ranges were selected based on preliminary laboratory evaluations and values reported in previous studies [26,28].

The response variables analyzed in the Taguchi experiment were the numbers of eggs, larvae, pupae, and adults produced of *D. suzukii*. These developmental parameters were used as indirect indicators of the effectiveness of each antimicrobial combination in suppressing microbial contamination while maintaining the suitability of the diet for insect development.

Twenty-seven diet formulations, based on the standard wheat-germ diet (i.e., the diet routinely used at the CNRCB) with all other ingredients and preparation procedures maintained but stripped of all antimicrobials, were supplemented according to the antimicrobial treatment combinations defined by the Taguchi L27 design (Figure 1) and prepared as previously described. Antimicrobial compounds were incorporated either directly into the dry ingredients before adding the unheated water (sodium benzoate, potassium sorbate, and sodium propionate) or dissolved in ethanol and added after the mixture had been boiled and cooled to room temperature (propyl paraben and triclosan). The resulting diet was dispensed into eight Petri dishes (5.4 cm diameter × 1.4 cm height) (i.e., replicates per treatment). This entire evaluation procedure was independently replicated three times.

To obtain experimental homogeneity, all experimental Petri dishes were exposed for 4 h inside a rearing cage (70 × 70 × 70 cm) containing 1000 *D. suzukii* adults (≤4 days old) to allow oviposition. Following exposure, each Petri dish was individually transferred to a Styrofoam cup (8.7 cm diameter × 11.3 cm height) covered with organza fabric. All dishes were carefully inspected to ensure that no adult flies remained inside. The numbers of eggs, larvae, pupae, and emerged adults were recorded at 1, 3, 10, and 15 days after oviposition, respectively. Eggs, larvae, and pupae were counted under a stereomicroscope, whereas adult emergence was recorded daily. Emerging adults were counted by opening each cup inside a rearing cage to prevent insect escapes.

### 2.4. Validation of the Taguchi-Optimized Antimicrobials

The predicted optimal concentrations of the Taguchi-selected antimicrobials were validated by incorporating them into the wheat germ diet and comparing their performance with that of standard rearing diets. Two candidate formulations were evaluated: (1) the theoretical optimum combination, obtained through the Taguchi Experimental Design Matrix optimization procedure, which was defined by the factor levels predicted to maximize overall biological performance, and (2) the best-performing pupae treatment, which produced the highest pupal yield in the Taguchi experiment. These formulations were compared with two control diets: (1) an internal control consisting of the standard wheat germ diet without any added antimicrobial compounds throughout the entire preparation process, and (2) an external control corresponding to the standard wheat-germ diet routinely used at the CNRCB, containing 4 g sodium benzoate, 1.2 g propyl paraben, 1.2 g potassium sorbate, 1.2 g sodium propionate, and 0.15 g triclosan per unit of diet [13,26,28].

The four wheat germ diet variants were prepared following the standard protocol described above, with antimicrobial compounds incorporated at the concentrations specified for each treatment. The resulting diet was dispensed into four transparent Petri dishes (i.e., replicates per treatment). This entire evaluation procedure was replicated four times.

The Petri dishes were randomly distributed between two *D. suzukii* rearing cages and simultaneously exposed to adult flies for 5 h to allow oviposition. After exposure, the number of eggs deposited in each dish was recorded using a stereomicroscope (Stemi 508, ZEISS, Jena, Germany). Egg hatch was assessed 48 h after oviposition, pupal production was recorded 10 d after oviposition, and the number of emerged adults was evaluated 14 d after oviposition. The total number of emerged adults and the sex ratio (female) were determined for each experimental unit.

All experimental units were maintained under controlled laboratory conditions of 23 ± 1 °C, 40 ± 5% RH, and a natural photoperiod ranging from 11:13 to 13:11 h (L:D), with an average of 12:12 h (L:D) throughout the study period.

### 2.5. Statistical Analysis

Statistical analyses were performed using Minitab v.20.3 (Minitab, LLC., State College, PA, USA). The software was used to generate the Taguchi L27 (3^5^) orthogonal array and to analyze the experimental data according to the Taguchi method [34]. For each treatment, the signal-to-noise (S/N) ratio and the mean number of eggs, larvae, pupae, and adults were calculated. The “larger-the-better” S/N criterion was used because maximizing pupal production was the primary objective of the study. The theoretical optimum concentration level of each antimicrobial was determined from the main effects plots by selecting the factor levels that simultaneously maximized both the S/N ratio and the arithmetic mean of the response variables.

In addition, Tukey’s multiple comparison test was applied to the results obtained from the L27 (3^5^) design to identify the treatment that produced the highest pupal yield. Pupal production was considered the primary response variable because each pupa represents a potential host for the parasitoid *T. drosophilae*.

To validate the predicted optimal formulation, differences among treatments in the number of eggs, larvae, pupae, and adults of *D. suzukii* were analyzed using one-way ANOVA, followed by Tukey’s multiple comparison tests. In all cases, statistical significance was determined at *p* ≤ 0.05, and normality of residuals and homogeneity of variances were verified before conducting the analyses. All figures were generated in R v.4.5 (R Core Team 2025) using the ggplot2 package [39,40].

## 3. Results

### 3.1. Antimicrobial Selection Process in the Wheat Germ Diet

**Figure 2:**
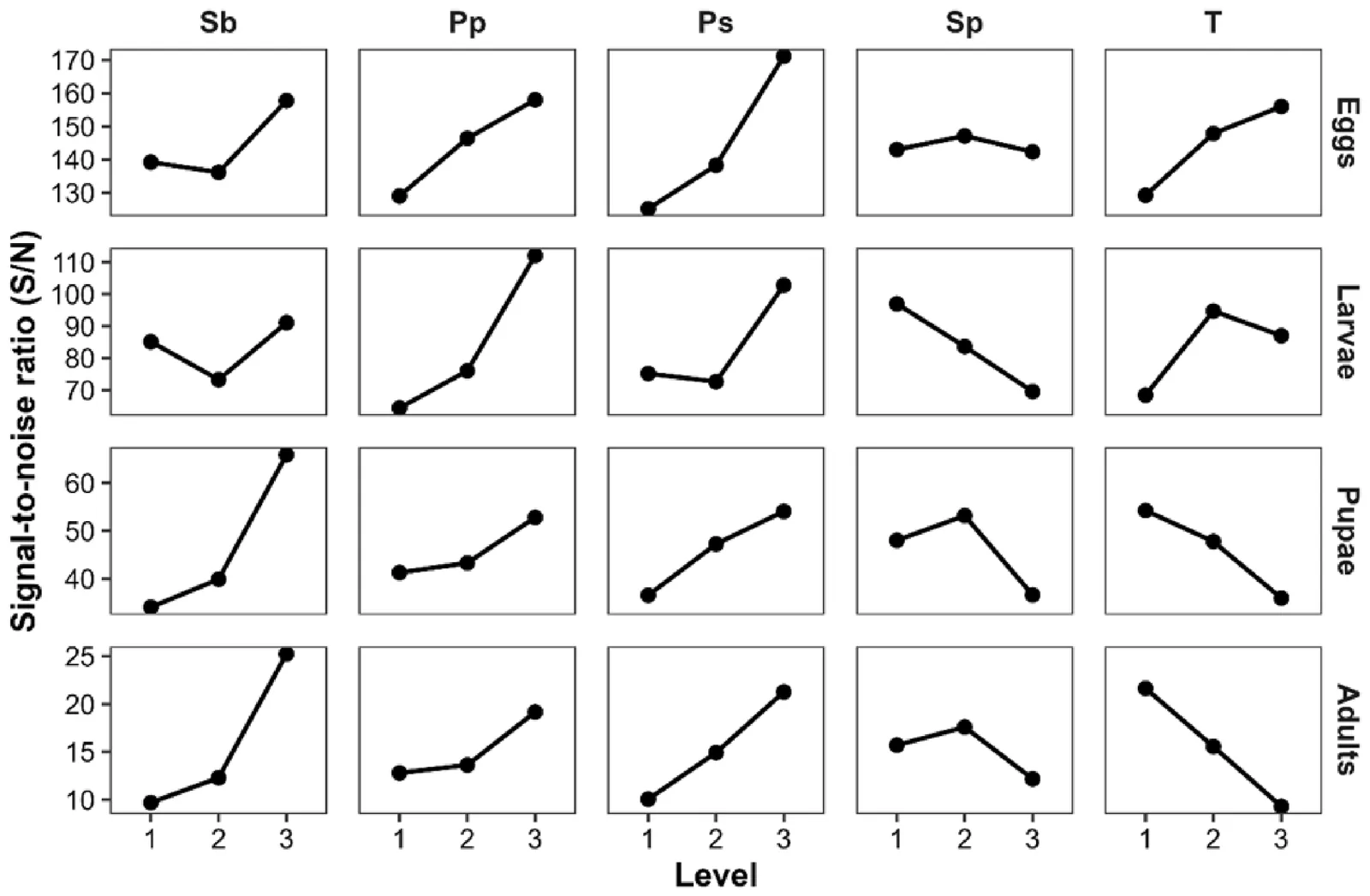
Main effects showing the Signal-to-noise (S/N) ratio of eggs, larvae, pupae, and adults of *Drosophila suzukii* on an artificial diet supplemented with five antimicrobials: sodium benzoate (Sb), propyl paraben (Pp), potassium sorbate (Ps), sodium propionate (Sp), and triclosan (T). Levels 1, 2, and 3 represent 0 g, 0.3 ± 0.1 g, and 1.2 ± 0.1 g, respectively, for Sb, Pp, Ps, and Sp; and 0 g, 0.075 ± 0.01 g, and 0.15 ± 0.01 g, respectively, for T. Data were analyzed using a Taguchi L27 (3^5^) orthogonal array design.

**Figure 3:**
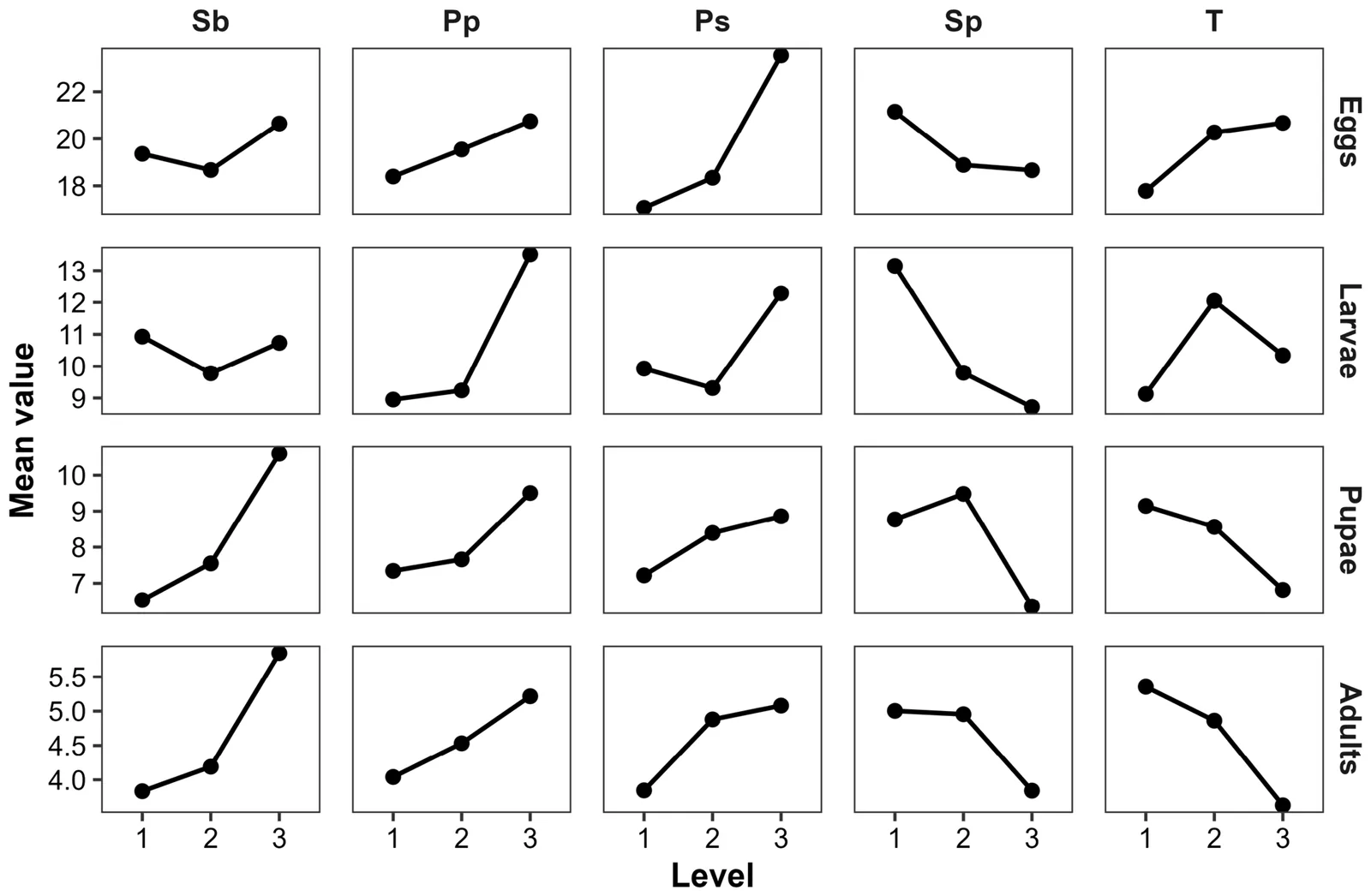
Main effects plot showing the arithmetic mean number of eggs, larvae, pupae, and adults of *Drosophila suzukii* on an artificial diet supplemented with five antimicrobials: sodium benzoate (Sb), propyl paraben (Pp), potassium sorbate (Ps), sodium propionate (Sp), and triclosan (T). Levels 1, 2, and 3 correspond to 0 g, 0.3 ± 0.1 g, and 1.2 ± 0.1 g, respectively, for Sb, Pp, Ps, and Sp; and to 0 g, 0.075 ± 0.01 g, and 0.15 ± 0.01 g, respectively, for T. Data were analyzed using a Taguchi L27 (3^5^) orthogonal array design.

According to the Taguchi L27 orthogonal array (3^5^), the antimicrobial concentrations that induced optimal oviposition (eggs) by *D. suzukii* females were L3, L3, L3, L1, and L2 of sodium benzoate, propyl paraben, potassium sorbate, sodium propionate, and triclosan, respectively. The best combination of antimicrobials for larval survival was L3, L3, L3, L1, and L2, respectively. For both pupae and adults, the best combination was L3, L3, L2, L1, and L1, respectively (Figure 2). The optimal level combinations were identified from the signal-to-noise ratios and confirmed using main effects plots of the arithmetic means (Figure 2 and Figure 3, respectively).

Considering all evaluated response variables collectively, the theoretically optimized antimicrobial combination was L3, L3, L2, L1, and L1, corresponding to 1.2 g L^−1^ of sodium benzoate, 1.2 g L^−1^ of propyl paraben, 0.3 g L^−1^ of potassium sorbate, 0 g L^−1^ of sodium propionate, and 0 g L^−1^ of triclosan, respectively [hereafter referred to as the Optimal Taguchi antimicrobial diet formulation (OTADF)].

### 3.2. Effect of Antimicrobial Treatments on Pupal Production

Pupal production differed significantly among the 27 treatments of the Taguchi L27 (3^5^) orthogonal array (one-way ANOVA: F = 3.96, df = 26, 621, *p* < 0.001; followed by Tukey’s HSD test; Table 2). Treatment 19 (1.2 g sodium benzoate, 0 g propyl paraben, 0.3 g potassium sorbate, 1.2 g sodium propionate, and 0 g triclosan) produced the highest number of pupae (20.1 ± 0.4), corresponding to an increase of 3.9 above the control without antimicrobials (T1) and a treatment-to-control ratio of approximately 4.9. In contrast, treatment 9 resulted in the lowest pupal production (3.0 ± 0.2). Treatments 26, 27, 17, and 13 also showed substantially higher pupal production than the control, with increases ranging from 2.1 to 2.8 above the control, corresponding to treatment-to-control ratios of approximately 3.1 to 3.8 (Table 2). The remaining treatments produced intermediate responses (Table 2).

### 3.3. Validation of the Optimal Taguchi Antimicrobial Diet Formulation

The validation assay of the optimized diets in *D. suzukii* rearing revealed no significant differences in oviposition among the four diets evaluated (one-way ANOVA: F = 2.28, df = 3, 60, *p* = 0.089). Mean oviposition was 53.28 ± 0.51, 47.73 ± 0.53, 39.58 ± 0.67, and 30.75 ± 0.22 eggs per Petri dish for the best pupae selected diet (T19), OTADF, the external control (CNRCB diet), and the internal control, respectively. Accordingly, all treatments belonged to the same statistical group (Table 3).

Regarding larval production, significant overall differences were detected among treatments (one-way ANOVA: F = 12.54, df = 3, 60, *p* < 0.0001). The best pupae selected diet (45.45 ± 0.47 larvae), OTADF (36.71 ± 0.57), and external control (CNRCB) (29.64 ± 0.71) yielded comparable numbers of larvae, whereas the internal control (without antimicrobials) produced significantly fewer larvae (12.47 ± 0.22) than the other treatments (Table 3).

In contrast, for the two most important production parameters (pupal formation and adult emergence), the best pupae selected diet (T19) and the OTADF performed similarly and significantly outperformed both control diets. These formulations produced the highest numbers of pupae (15.78 ± 0.19 and 12.07 ± 0.09, respectively) and adults (10.84 ± 0.12 and 8.49 ± 0.07, respectively) (one-way ANOVA: F = 28.26, df = 3, 60, *p* < 0.0001). Relative to the internal control, the best pupae selected diet (T19) increased pupal production by 280.2% and adult emergence by 204.5%, whereas the OTADF increased pupal production by 190.8% and adult emergence by 138.5% (Table 3).

Overall rearing efficiency was assessed by calculating the cumulative loss from egg to adult (Table 3). The best pupae selected diet (T19) exhibited the lowest total loss (79.7%), corresponding to an egg-to-adult ratio of 4.92 relative to the initial egg load, followed by the OTADF (82.2%), corresponding to a ratio of 5.62. Although no formal statistical testing was applied to this overall cumulative metric, these numerical values indicate enhanced rearing efficiency for T19 and OTADF compared to both the external control (86.1% and egg-to-adult ratio of 7.17) and internal control (88.4% and a ratio of 8.64).

## 4. Discussion

The results of this study, although microbial populations were assessed indirectly through host performance, contribute to the development of a cost-effective rearing system based on inexpensive ingredients and straightforward procedures for mass rearing of *D. suzukii*, thereby facilitating its adoption by laboratories and mass-rearing facilities operating under limited financial resources. Such an approach is particularly relevant for biological control programs, where reducing production costs while maintaining insect quality is essential for large-scale implementation by both public institutions and private enterprises [28,30].

In this context, the Taguchi orthogonal array design proved to be a valuable tool for optimizing antimicrobial combinations in the artificial diet. By substantially reducing the number of experimental treatments required compared with a full factorial design, the Taguchi approach enabled the simultaneous evaluation of multiple factors and their levels while efficiently identifying optimal treatment combinations [33,37]. This methodology not only minimizes experimental costs and labor requirements but also enhances decision-making during the optimization process, making it especially useful for insect mass-rearing programs where resources and time are often limited.

### 4.1. Taguchi-Selected Formulations for Colony Performance and Pupal Production

The Taguchi orthogonal array identified an antimicrobial combination that significantly improved both colony performance and pupal production of *D. suzukii* reared on a low-cost artificial diet. The optimal Taguchi antimicrobial diet formulation (OTADF), consisting of 1.2 g L^−1^ sodium benzoate, 1.2 g L^−1^ propyl paraben, 0.3 g L^−1^ potassium sorbate, and no sodium propionate or triclosan, effectively suppressed microbial contamination while maintaining, and in some cases improving, key biological parameters of the insect colony.

Among the 27 antimicrobial combinations evaluated, Treatment 19 yielded the highest pupal production, corresponding to an increase of 3.9 above the antimicrobial-free control and a treatment-to-control ratio of approximately 4.9. The validation assay further demonstrated that both the OTADF and Treatment 19 consistently outperformed the internal and external control diets in terms of pupal and adult production. Improvements of up to 280% in pupal yield and 204% in adult emergence were recorded relative to the internal control, highlighting the substantial impact of antimicrobial optimization on colony productivity.

These findings are particularly relevant for the mass production of the pupal parasitoid *T. drosophilae*, for which host pupae represent the critical developmental resource. Although Treatment 19 produced the highest numerical pupal yield, its performance was not statistically different from that of the OTADF. Considering its biological performance comparable to that of Treatment 19 and its derivation from the overall optimization process, the OTADF represents the most practical and efficient option for routine colony maintenance and large-scale production. The adoption of a single standardized formulation may also simplify diet preparation, improve operational consistency, and facilitate technology transfer among insect-rearing facilities.

### 4.2. Synergistic Selection and Efficiency of the Antimicrobial Taguchi Matrix

The study initially evaluated five antimicrobials with different spectra of activity and modes of action to achieve broad protection against microbial contamination. However, the Taguchi analysis revealed that not all compounds contributed positively to this specific wheat germ-based diet. The method efficiently demonstrated that only three antimicrobials were necessary, leading to the exclusion of sodium propionate and triclosan from the final optimized formulation. The optimal combination consisted of higher concentrations (L3) of sodium benzoate and propyl paraben combined with a moderate level (L2) of potassium sorbate. This triad provides robust, broad-spectrum antimicrobial activity through complementary mechanisms, including membrane disruption, enzyme inhibition, and cytoplasmic acidification (Table 1), while reducing the total chemical load and operational costs [37,40].

This multi-component strategy, inspired by the principles outlined by Cohen [29,30] (2015, 2018), was designed to (i) broaden the antimicrobial spectrum against fungi, bacteria, and yeasts; (ii) minimize the risk of resistance development; and (iii) enhance overall efficacy at lower individual concentrations. Although conventional artificial diets generally rely on only one to three antimicrobial agents [41], the present study explored a broader combinatorial framework to identify the most effective antimicrobial matrix. The resulting synergy provided superior microbial control without compromising insect performance. Oviposition and larval survival remained comparable to or higher than those observed with the external standard control (CNRCB), while cumulative egg-to-adult mortality was substantially reduced (up to a ratio of 4.92 in Treatment 19). Taken together, these findings indicate that a carefully designed multi-targeted antimicrobial combination may outperform conventional preservative strategies, providing a more stable, efficient, and biologically compatible environment for the mass rearing of *D. suzukii* and the subsequent production of its parasitoid *T. drosophilae*.

### 4.3. Advantages of Using the Optimal Taguchi Antimicrobial Diet Formulation

The optimized Taguchi antimicrobial diet offers significant improvements in three main aspects. First, the OTADF substantially reduces the quantity of antimicrobials required compared with the standard CNRCB diet. Although a detailed economic analysis was not conducted, this reduction is expected to generate meaningful cost savings that may accumulate into substantial annual economic benefits in a continuous mass-rearing system [29,30]. Second, using fewer compounds and lower concentrations simplifies diet preparation, reduces processing time, and minimizes handling errors, thereby improving operational efficiency. Third, by building upon the low-cost, agar-free, wheat germ-based diet developed in Mexico, this optimization further enhances economic viability and accessibility. Agar-free diets also reduce the generation of biohazardous waste while eliminating the need for expensive infrastructure and specialized disposal procedures. As a result, this OTADF represents a practical and cost-effective alternative for berry-producing regions in developing countries, where high implementation costs often constrain the large-scale production of *T. drosophilae* for the biological control of *D. suzukii*.

### 4.4. Implications for Integrated Pest Management (IPM) of D. suzukii

The use of a low-cost diet greatly facilitates scalable production, particularly of parasitoids against *D. suzukii* [42]. By significantly lowering operational costs, this rearing system enables the implementation of augmentative release programs in berry orchards. This approach not only reduces reliance on chemical insecticides but also promotes the long-term sustainability of soft-fruit production [24,43].

In this context, within a cyclical insect production system, even modest improvements in rearing efficiency can generate considerable cumulative benefits over time [30]. In the case of the CNRCB mass-rearing program, which operates on a continuous three-day production cycle, the enhanced microbial control provided by the optimized formulation is expected to substantially increase pupal output. The synergy between the Taguchi-optimized antimicrobial matrix and this continuous rearing system is projected to generate thousands of additional high-quality parasitoids per year, thereby strengthening regional IPM efforts against this invasive pest.

### 4.5. Appropriateness of the Taguchi Method for This Study

Although general guidelines exist for the incorporation of antimicrobials in artificial insect diets, the optimal concentrations and combinations must always be determined experimentally for each specific rearing system and target species. This is due to complex interactions among the diet matrix, environmental conditions, and insect physiology, which can significantly influence both antimicrobial efficacy and overall insect performance [44].

In this study, the Taguchi orthogonal array design (L27 = 3^5^) was selected as a robust and efficient statistical tool to identify the optimal antimicrobial combination with minimal experimental effort. The suitability of this approach becomes particularly evident when compared to traditional methods. For example, evaluating five antimicrobials at three concentration levels would have required 243 experimental runs using a full factorial design [33]. By leveraging the orthogonality of the Taguchi method, reliable optimization data were obtained with only 27 treatments. This substantial reduction in experimental workload allowed efficient screening of multiple inhibitors while preserving high statistical power. Furthermore, the use of the Signal-to-Noise (S/N) ratio ensured that the final formulation was not only highly productive but also robust against environmental variability, such as the microbial contamination pressures commonly encountered in mass-rearing facilities. These results clearly demonstrate the suitability and practical value of the Taguchi method for the development and continuous improvement of artificial diets in applied entomology.

### 4.6. Limitations and Future Perspectives

Although the confirmation assay yielded robust results and provided a practical solution to the persistent microbial contamination issues at the CNRCB, this study acknowledges certain limitations that highlight important avenues for future research. First, the effectiveness of the different antimicrobial concentrations on microbial populations was assessed indirectly through host performance (egg, larval, pupal, and adult yields) rather than through direct microbial quantification. Future studies should incorporate direct microbial assays, such as the quantification of colony-forming units (CFUs), to more precisely characterize the inhibitory dynamics of the optimized matrix.

This study identified an artificial diet that increased *D. suzukii* pupal production. Because host availability is a key limiting factor in the mass production of *T. drosophilae*, these findings represent an important step toward improving parasitoid production, although the direct effects of the OTADF on the biological performance of the parasitoid were not evaluated. Future research should assess its impact on adult longevity, fecundity, parasitism rate, and dispersal capacity. Likewise, pupal quality was evaluated solely through numerical yield, without measuring physical pupal traits (such as weight or dimensions); therefore, such complementary studies remain necessary to fully validate mass-rearing applications. Additional studies should also assess diet stability under commercial insectary conditions and investigate potential antimicrobial residues in pupae and their possible impacts on the parasitoid or non-target organisms. Such data will be critical for obtaining regulatory approval and for ensuring the acceptance of this technology among organic producers and IPM practitioners.

### 4.7. Context Within Global Artificial Diet Development for D. suzukii

Several research groups are actively developing and refining artificial diets for *D. suzukii* to improve the mass production of its parasitoids, particularly *T. drosophilae*. However, most of these formulations continue to rely heavily on agar as a gelling agent [45,46]. Although some studies have proposed agar-free diets, these formulations have generally been developed for specific experimental purposes and have rarely been adopted or cited in subsequent research [47,48,49].

In contrast, our low-cost, wheat germ-based diet has demonstrated continuity and scientific impact over time. It has supported research by independent academic groups, resulting in publications in both national and international scientific journals, and it has also been incorporated into production manuals and technical data sheets for *T. drosophilae* [26,27,28,50,51,52,53,54,55]. By publishing this improved formulation with the optimized antimicrobial matrix, updated and more efficient protocols are now available to the international scientific community. This cumulative body of work consolidates a robust, low-cost rearing technology for both the pest *D. suzukii* and its biological control agent *T. drosophilae*.

### 4.8. Strategic Impact of This Research on the IPM of D. suzukii

This advancement strengthens the accessible mass-rearing of *T. drosophilae* as a viable and sustainable strategy for the suppression of *D. suzukii*, particularly in strawberry and other berry production systems in resource-constrained regions [56]. Since its first detection in Mexico in 2011, this invasive pest has spread rapidly. By February 2020, it was reported across five key states: Baja California, Colima, Jalisco, Guanajuato, and Michoacán [12,13]. The results obtained in this study, particularly the reduction in microbial contamination and the resulting increase in pupal yield, provide a critical tool for the CNRCB to scale up parasitoid production. By reducing dependence on costly inputs and complex infrastructure, the optimized diet promotes the broader adoption of biological control within sustainable IPM frameworks [27,57,58].

The ability to efficiently mass-produce and release *T. drosophilae* is essential to safeguard the economic stability of the berry industry in Mexico, which was valued at USD 2.106 billion in 2024, and generates approximately half a million direct jobs [24,43,55,58]. Therefore, implementing these optimized rearing protocols not only addresses a technical entomological challenge but also contributes to preserving the livelihoods dependent on one of the country’s most significant agricultural exports.

## 5. Conclusions

The Taguchi experimental design proved to be an efficient and powerful statistical tool for the development and optimization of artificial diets in applied entomology. The proposed antimicrobial combination (1.2 g L^−1^ of sodium benzoate, 1.2 g L^−1^ of propyl paraben, 0.3 g L^−1^ of potassium sorbate) represents a significant step toward accessible and economical mass-rearing of *D. suzukii.* Because host availability is a key limiting factor in the mass production of *T. drosophilae*, these findings also represent an important step toward improving parasitoid production. This technology offers a viable and sustainable approach for improving *D. suzukii* mass rearing, thereby opening new opportunities for furthering the biological control of one of the world’s most destructive berry pests, particularly in resource-limited settings.

## Figures and Tables

**Table 1:** Characteristics and antimicrobial activities of five compounds used to control microbial contamination in an artificial diet for *Drosophila suzukii*.

Antimicrobials	Compound Class *	CAS No.	Target Microorganisms	Primary Mode of Action
**Sodium benzoate**	Organic acid salt	532-32-1	Fungi	Damages the cell membrane, disrupts intracellular pH homeostasis, and interferes with nutrient transport and energy production
**Propyl paraben**	Paraben (p-hydroxybenzoic acid ester)	94-13-3	Fungi and bacteria	Alters cytoplasmic membrane permeability, disrupts membrane-associated transport processes, and inhibits essential enzymatic activities
**Potassium sorbate**	Organic acid salt	24634-61-5	Fungi, yeasts, and some bacteria	Inhibits metabolic enzymes, disrupts oxidative phosphorylation, and compromises membrane integrity.
**Sodium propionate**	Organic acid salt	137-40-6	Fungi	Interferes with protein synthesis, inhibits enzymes involved in glucose metabolism, and disrupts intracellular pH regulation
**Triclosan**	Chlorinated phenolic compound	3380-34-5	Bacteria	Inhibits fatty acid biosynthesis by targeting enoyl-acyl carrier protein reductase, thereby impairing membrane synthesis and cell growth.

Source: Information on the mode of action was interpreted from Schweizer [31] (2001) and Davidson et al. [32] (2021). * Organic acid salts are salts derived from weak organic acids. In the present study, sodium benzoate is a salt of benzoic acid, potassium sorbate is a salt of sorbic acid, and sodium propionate is a salt of propionic acid. Parabens are esters of p-hydroxybenzoic acid, whereas chlorinated phenolic compounds are halogenated aromatic compounds with antimicrobial properties.

**Table 2:** Pupal production of *Drosophila suzukii* on artificial diets supplemented with different antimicrobial treatments using a Taguchi L27 (3^5^) orthogonal array.

Treatment	Number of Pupae	Increase Above Control *
**1**	4.1 ± 0.3 de	
**2**	6.3 ± 0.3 bcde	0.539
**3**	6.4 ± 0.3 bcde	0.566
**4**	8.4 ± 0.3 abcde	1.041
**5**	8.5 ± 0.4 abcde	1.063
**6**	5.9 ± 0.3 bcde	0.427
**7**	11.3 ± 0.4 abcde	1.76
**8**	6.9 ± 0.2 bcde	0.686
**9**	3.0 ± 0.2 e	−0.267
**10**	6.1 ± 0.4 bcde	0.499
**11**	5.3 ± 0.2 bcde	0.29
**12**	4.7 ± 0.2 bcde	0.144
**13**	12.9 ± 0.4 abcd	2.149
**14**	7.6 ± 0.3 bcde	0.842
**15**	5.5 ± 0.2 bcde	0.332
**16**	7.6 ± 0.3 bcde	0.843
**17**	13.2 ± 0.3 abcd	2.217
**18**	7.4 ± 0.3 bcde	0.815
**19**	20.1 ± 0.4 a	3.913
**20**	9.9 ± 0.4 abcde	1.414
**21**	7.5 ± 0.3 bcde	0.84
**22**	6.1 ± 0.4 bcde	0.484
**23**	6.2 ± 0.2 bcde	0.522
**24**	9.3 ± 0.4 abcde	1.269
**25**	10.0 ± 0.3 abcde	1.449
**26**	15.6 ± 0.3 ab	2.817
**27**	14.3 ± 0.3 abc	2.496

Means (±SE) followed by the same letter within a column are not significantly different (one-way ANOVA followed by Tukey’s HSD test, *p* ≤ 0.05). Treatment 1 represents the control diet without antimicrobials. * Increase above the antimicrobial-free control (T1), calculated relative to the control value.

**Table 3:** Effects of antimicrobial diet formulations selected by the Taguchi method on oviposition, larval production, pupal formation, and adult emergence of *Drosophila suzukii*.

Diet	No. Eggs	Eggs → Larvae Reduction (%)	No. Larvae	Larvae → Pupae Reduction (%)	No. Pupae	Pupae → Adults Reduction (%)	No. Adults	Total Loss (%)	Egg-to-Adult Ratio
Best pupae selected (T19)	53.28 ± 0.51 a	14.7	45.45 ± 0.47 a	65.3	15.78 ± 0.19 a	31.3	10.84 ± 0.12 a	79.7	4.92
OTADF	47.73 ± 0.53 a	23.1	36.71 ± 0.57 a	67.1	12.07 ± 0.09 a	29.7	8.49 ± 0.07 a	82.2	5.62
External control (CNRCB)	39.58 ± 0.67 a	25.1	29.64 ± 0.71 ab	75.2	7.34 ± 0.19 b	24.8	5.52 ± 0.13 b	86.1	7.17
Internal control (T1)	30.75 ± 0.22 a	59.4	12.47 ± 0.22 b	66.7	4.15 ± 0.16 c	14.2	3.56 ± 0.11 c	88.4	8.64

Means (±SE) followed by the same letter within a column are not significantly different (one-way ANOVA followed by Tukey’s HSD test, *p* ≤ 0.05). OTADF = optimal Taguchi antimicrobial diet formulation; external control = standard wheat-germ-based diet with all other ingredients, including the regular antimicrobials; internal control = standard wheat-germ-based diet prepared without any added antimicrobial agents throughout the entire preparation process.

## Data Availability

Data are available from the corresponding author upon reasonable request.

## Abbreviations

The following abbreviations are used in this manuscript:

IPM	Integrated pest management
CNRCB	National Reference Center for Biological Control
DGSV	General Directorate of Plant Health
SENASICA	National Agro-alimentary Health, Safety and Quality Service
OTADF	Optimal Taguchi antimicrobial diet formulation
